# Differential Regulation of Allergic Airway Inflammation by Acetylcholine

**DOI:** 10.3389/fimmu.2022.893844

**Published:** 2022-05-27

**Authors:** Luke B. Roberts, Rita Berkachy, Madina Wane, Dhiren F. Patel, Corinna Schnoeller, Graham M. Lord, Kleoniki Gounaris, Bernhard Ryffel, Valerie Quesniaux, Matthew Darby, William G. C. Horsnell, Murray E. Selkirk

**Affiliations:** ^1^ Department of Life Sciences, Imperial College London, London, United Kingdom; ^2^ School of Immunology and Microbial Sciences, King’s College London, Great Maze Pond, London, United Kingdom; ^3^ National Heart and Lung Institute, Imperial College London, London, United Kingdom; ^4^ Faculty of Biology, Medicine and Health, University of Manchester, Manchester, United Kingdom; ^5^ Laboratory of Molecular and Experimental Immunology and Neurogenetics, UMR 7355, CNRS-University of Orleans and Le Studium Institute for Advanced Studies, Rue Dupanloup, Orléans, France; ^6^ Institute of Infectious Disease and Molecular Medicine, University of Cape Town, Cape Town, South Africa; ^7^ College of Medical and Dental Sciences, University of Birmingham, Birmingham, United Kingdom

**Keywords:** lung, alternaria, ILC2, neutrophil, eosinophil, inflammation, chemokine, acetylcholine

## Abstract

Acetylcholine (ACh) from neuronal and non-neuronal sources plays an important role in the regulation of immune responses and is associated with the development of several disease pathologies. We have previously demonstrated that group 2 innate lymphoid cell (ILC2)-derived ACh is required for optimal type 2 responses to parasitic infection and therefore sought to determine whether this also plays a role in allergic inflammation. *Rora* ^Cre+^ *Chat* ^LoxP^ mice (in which ILC2s cannot synthesize ACh) were exposed to an allergenic extract of the fungus *Alternaria alternata*, and immune responses in the airways and lung tissues were analyzed. Airway neutrophilia and expression of the neutrophil chemoattractants CXCL1 and CXCL2 were enhanced 24 h after exposure, suggesting that ILC2-derived ACh plays a role in limiting excessive pulmonary neutrophilic inflammation. The effect of non-selective depletion of ACh was examined by intranasal administration of a stable parasite-secreted acetylcholinesterase. Depletion of airway ACh in this manner resulted in a more profound enhancement of neutrophilia and chemokine expression, suggesting multiple cellular sources for the release of ACh. In contrast, depletion of ACh inhibited *Alternaria*-induced activation of ILC2s, suppressing the expression of IL-5, IL-13, and subsequent eosinophilia. Depletion of ACh reduced macrophages with an alternatively activated M2 phenotype and an increase in M1 macrophage marker expression. These data suggest that ACh regulates allergic airway inflammation in several ways, enhancing ILC2-driven eosinophilia but suppressing neutrophilia through reduced chemokine expression.

## Introduction

Acetylcholine (ACh) is best known as a neurotransmitter, but in recent years, it has been increasingly implicated as an important signaling molecule in the immune system. It was first identified as a negative regulator of inflammatory cytokine production by macrophages in what is termed the cholinergic anti-inflammatory pathway (1), and was subsequently demonstrated to influence the trafficking, activation and effector functions of T cells during infection (2, 3). Our studies have indicated that ACh is necessary for effective control of the parasitic nematode *Nippostrongylus brasiliensis*, in part by regulating physiological responses such as smooth muscle contraction, but also contributing to adaptive immunity by signaling through the M3 muscarinic acetylcholine receptor (mAChR) to promote cytokine production by CD4^+^ T cells (2). We and others recently determined that group 2 innate lymphoid cells (ILC2s) are a major source of ACh during nematode infection and that genetic disruption of ACh synthesis by ILC2s renders mice more susceptible to infection, with reduced barrier responses in the lungs and gut (4, 5).

ACh is the major parasympathetic neurotransmitter in the airways, and excessive cholinergic signaling contributes to the pathology of asthma and chronic obstructive pulmonary disease (COPD) by promoting bronchoconstriction, mucus production, and airway remodeling (6). ACh also appears to play a pro-inflammatory role, which impacts on the pathology of lung disease (7). Neurons are a major source of ACh in the lungs, but it is also synthesized and released by non-neuronal cells, namely, pulmonary epithelial and immune cells, including ILC2s (4, 8–10).

Asthmatic inflammation is characterized by increased type 2 cytokines and eosinophilia in the lungs. ILC2s can be activated by release of epithelial alarmins including interleukin (IL)-33, and thus act as an important link between the innate and the adaptive immune system in the initiation of allergic inflammation (11). Because we had identified ILC2-derived ACh as an important factor in promoting barrier responses to nematode infection, we were interested in examining whether this played a role in the initiation of allergic inflammation in the lungs. Intranasal dosing of mice with an extract of the fungal mold *Alternaria alternata*, a common environmental aeroallergen, induces rapid release of interleukin (IL)-33 into the airway lumen, although the initial trigger appears to be the release of adenosine triphosphate (ATP) from epithelial cells (12). ILC2s produce large amounts of IL-5 and IL-13 in response to activation with IL-33, resulting in eosinophilia (13) and this has been adopted as a robust model for innate induction of airway inflammation (14).

In this study, we demonstrate that *Rora* ^Cre+^ *Chat* ^LoxP^ mice, in which ILC2s cannot synthesize ACh, show enhanced neutrophilia in the airways following acute exposure to an allergenic extract of *A. alternata*. More profound neutrophilia was observed in wild-type mice following non-selective enzymatic depletion of ACh in the airways, which was accompanied by increased expression of neutrophil chemokines and a reduction of macrophages with an alternatively activated M2 phenotype. Non-selective depletion of ACh in the airways also strongly inhibited ILC2 activation, cytokine expression, and associated eosinophilia.

## Results

### ILC2-Derived Acetylcholine Inhibits Neutrophilia Following Exposure to *Alternaria* Allergen Extract

It is unclear how cholinergic signaling regulates pulmonary immune responses during type 2-dominated airway inflammation. We have recently shown that ILC2s are significant producers of ACh following alarmin-mediated activation in the context of type 2 immunity against parasitic nematode infection and in response to *A. alternata* extract (4). During *Alternaria*-induced inflammation, serine protease activity releases IL-33 from airway epithelial cells (15), which activates many immune cell types, including ST2^+^ ILC2s. ILC2s are central mediators of type 2 inflammation, including eosinophilia (16), and we therefore explored whether ILC2-derived ACh was required for the generation of this response.

**Figure 1:**
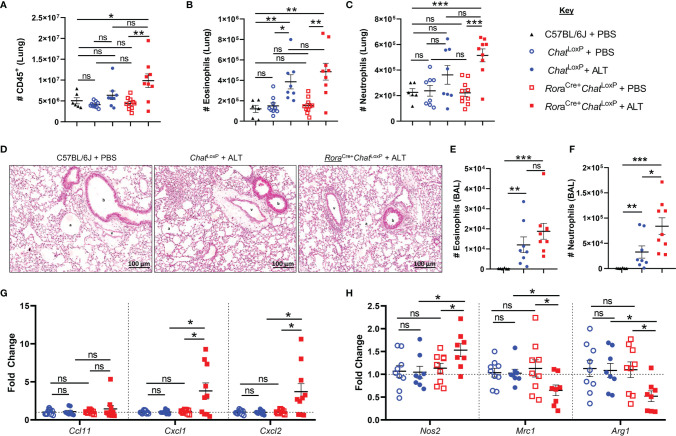
Granulocyte responses in the lung and airways of Rora^Cre+^Chat^LoxP^ mice following acute fungal allergen challenge. Lung and airway leucocytes were analyzed from C57BL/6J, Chat^LoxP^, and Rora^Cre+^Chat^LoxP^ mice at baseline, and following Alternaria alternata allergen extract (ALT) exposure in Chat^LoxP^ and Rora^Cre+^Chat^LoxP^ mice, 24 h after intranasal dosing. **(A)** Number of CD45^+^ leucocytes per lung. **(B)** Number of eosinophils in the lung. **(C)** Number of neutrophils in the lung. **(D)** Representative H&E stained lung sections. b, bronchiole; v, blood vessel; a, alveolus. **(E)**. Number of eosinophils in the airways. **(F)** Number of neutrophils in the airways. **(G)** Fold change of Ccl11 (eotaxin-1), Cxcl1, and Cxcl2 transcript expression in total lung tissue, compared to baseline Chat^LoxP^. **(H)** Fold change of M1 macrophage marker Nos2 (inducible nitric oxide synthase, iNOS) and M2 macrophage markers Mrc1 (mannose receptor c-type 1, CD206) and Arg1 (arginase 1) transcript expression in total lung tissue, compared to baseline Chat^LoxP^. Data points represent individual animals. Data show pooled data points from 2 independent experiments with n = 3 C57BL/6J mice per group and n = 4–5 Rora^Cre+^Chat^LoxP^ and Chat^LoxP^ mice per experiment. Samples from untreated Chat^LoxP^ and Rora^Cre+^Chat^LoxP^ were collected on different days to C57BL/6J mice and ALT treated Chat^LoxP^ and Rora^Cre+^Chat^LoxP^, using animals from the same generations/litters and parents for both data sets. *p <0.05, **p <0.01, ***p <0.001, ns, non-significant difference (p > 0.05).

Administration of *Alternaria* to *Rora* ^Cre+^ *Chat* ^LoxP^ animals resulted in increased numbers of CD45^+^ cells in the lungs 24 h post-exposure (**Figure 1A** **).** The numbers of eosinophils and neutrophils in the lung tissue at baseline were comparable between *Rora* ^Cre+^ *Chat* ^LoxP^, *Chat* ^LoxP^, and C57BL/6J (WT) mice (**Figures 1B, C**). Exposure of *Rora* ^Cre+^ *Chat*LoxP and *Chat* ^LoxP^ mice to *Alternaria* resulted in a comparable eosinophil influx to lung tissue (**Figure 1B**). Although *Rora* ^Cre+^ *Chat* ^LoxP^ animals showed a trend toward increased numbers of neutrophils in the lungs, this was not significantly different from control groups at this timepoint (**Figure 1C**). Histologically, lung tissue appeared more inflamed in *Alternaria*-treated *Rora* ^Cre+^ *Chat* ^LoxP^ and *Chat* ^LoxP^ mice relative to untreated controls (**Figure 1D**). In the airways, alveolar macrophages (AM) were the dominant cell type in WT control mice, whereas significantly increased eosinophils and neutrophils were observed in *Alternaria* exposed *Rora* ^Cre+^ *Chat* ^LoxP^ and *Chat* ^LoxP^ genotypes (**Supplemental Figure 1** and **Figures 1E, F**) as expected (14, 17). Airway eosinophil numbers were comparable between *Alternaria*-exposed genotypes (**Figure 1E**), but more neutrophils were observed in *Rora* ^Cre+^ *Chat* ^LoxP^ mice (**Figure 1F**), indicating a role for ILC2-derived ACh in regulating neutrophil influx following exposure to *Alternaria*.

Analysis of transcript expression for chemokines involved in granulocyte trafficking revealed that eotaxin-1 (*Ccl11*) was unaffected in *Rora* ^Cre+^ *Chat* ^LoxP^ and *Chat* ^LoxP^ genotypes relative to WT mice and was also unaffected by exposure to *Alternaria* (**Figure 1G**). Transcripts for the neutrophil-attractant chemokines CXCL1/CXCL2 were also similar in untreated *Rora* ^Cre+^ *Chat* ^LoxP^ and *Chat* ^LoxP^ mice but were significantly elevated in *Rora* ^Cre+^ *Chat* ^LoxP^ mice following exposure to *Alternaria* (**Figure 1G**).

Proinflammatory macrophages with a classical ‘M1’ profile are key producers of neutrophil chemoattractants including CXCL1 and CXCL2, whereas alternatively activated or ‘M2’ macrophages do not characteristically express these molecules (18, 19). Expression of the M1 marker *Nos2* (inducible nitric oxide synthase, iNOS) was increased in the total lung tissue of *Rora* ^Cre+^ *Chat* ^LoxP^ mice, while the M2 markers *Mrc1* (mannose receptor C-type 1, CD206) and *Arg1* (arginase 1) were downregulated following *Alternaria* exposure (**Figure 1H**). These data suggest that increased neutrophil infiltration following *Alternaria* exposure might result from an M1 macrophage bias in the absence of ILC2-derived ACh.

**Figure 2:**
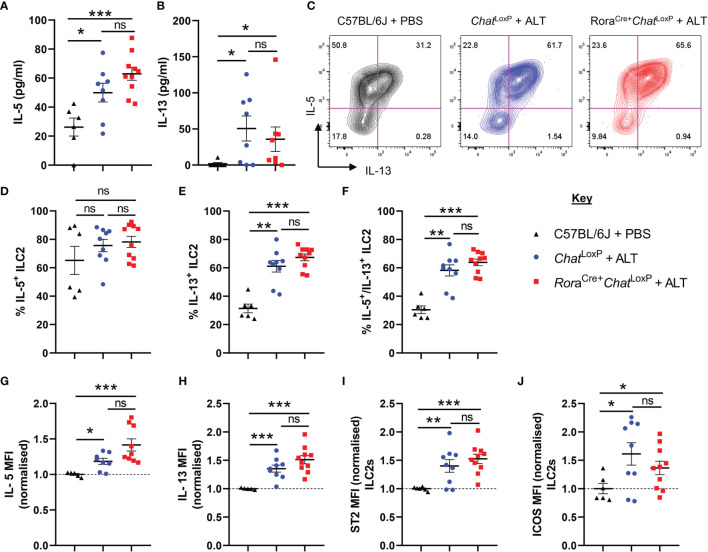
*ILC2 responses of Rora^Cre+^Chat^LoxP^ mice following acute fungal allergen challenge*. Pulmonary ILC2s were analysed from PBS-treated C57BL/6J mice and *Alternaria alternata* allergen extract (ALT) treated-Chat^LoxP^ and *Rora* ^Cre+^ *Chat* ^LoxP^ mice 24 h after intranasal dosing. **(A)** IL-5 and **(B)** IL-13 expression in the airways (bronchoalveolar lavage) as determined by ELISA. **(C)** Representative flow cytometry plots for IL-5 and IL-13 expression by pulmonary ILC2s from the indicated mouse strains and treatment groups. Numbers on the plots indicate the proportion of the parent ILC2 population for each gate. **(D)** Proportion of IL-5^+^ ILC2s. **(E)** Proportion of IL-13^+^ ILC2s. **(F)** Proportion of IL-5^+^IL-13^+^ ILC2s**. (G–J)** Mean fluorescence intensity (MFI) of **(G)** IL-5 staining for IL-5^+^ ILC2s **(H)** IL-13 staining for IL-13^+^ ILC2s. **(I)** ST2 staining for ILC2s. **(J)** ICOS staining for ILC2s. MFI data **(G–J)** are normalized to the mean of PBS treated C57BL/6J control values. Data points represent individual animals. Data show pooled data points from 2 independent experiments with n = 3 C57BL/6J mice per group and n = 4–5 *Rora* ^Cre+^ *Chat* ^LoxP^ and *Chat* ^LoxP^ mice per experiment. *p <0.05, **p <0.01, ***p < 0.001, ns, non-significant difference (*p >*0.05).

Analysis of IL-5 and IL-13 in bronchoalveolar lavage (BAL) fluid confirmed that dosing with *Alternaria* promoted a type 2 immune response. However, removal of the capacity of ILC2s to synthesize ACh did not impact the overall type 2 cytokine environment of the airways (**Figures 2A, B**). Expression of IL-5 and IL-13 (**Supplemental Figure 2A**, **Figures 2C–H**) and the activation markers ICOS or ST2 were not further altered in pulmonary ILC2s from *Rora* ^Cre+^ *Chat* ^LoxP^ mice (**Figures 2I, J**). Lung CD4^+^ T cells were also analysed as they are also a potential source of type 2 cytokines, can express RORα and are known producers of ACh (20). However, as expected given the acute nature of the model, expression of IL-5 and IL-13 by total CD4^+^ T cells (**Supplemental Figures 2B–G**) was extremely limited, and no differences between the genotypes were observed. A small proportion of total CD4^+^ T cells displayed a Th2 phenotype (Gata3^+^ST2^+^) which was unaltered in the lungs of *Alternaria*-treated mice (**Supplemental Figures 2B, H**), but again there were no significant differences between *Rora* ^Cre+^ *Chat* ^LoxP^ and *Chat* ^LoxP^ genotypes.

### Enzymatic Depletion of Airway Acetylcholine Prevents Eosinophilia But Exacerbates Neutrophilia Following Acute Exposure to *Alternaria* Allergen Extract

ILC2-derived ACh did not appear to greatly influence the onset of the type 2 immune response in the brief 24-hour timeframe of this model. However, ILC2s are only one cell type capable of ACh synthesis in the lung. We thus examined the effect of broader, non-selective depletion of ACh using a stable secretory acetylcholinesterase (AChE) from *N. brasiliensis* (21) and a catalytically inactive form of the enzyme generated by site-directed mutagenesis (22). The enzymatic activity of the preparations was confirmed by in-gel staining (**Supplemental Figure 3A**) and Ellman’s assay, which also confirmed that no inhibitors of AChE activity were present in the *Alternaria* extracts (**Supplemental Figure 3B**). Given our observations in *Rora* ^Cre+^ *Chat* ^LoxP^ mice, we were particularly interested in examining the neutrophilic response following ACh depletion. Therefore, we used BALB/c mice, which have a dominant early neutrophilic response to *Alternaria* exposure compared with C57BL/6 mice, which are more eosinophil-dominant (17). The enzymes were co-administered intranasally with *Alternaria*, as shown in **Supplemental Figure 4A**.

**Figure 3:**
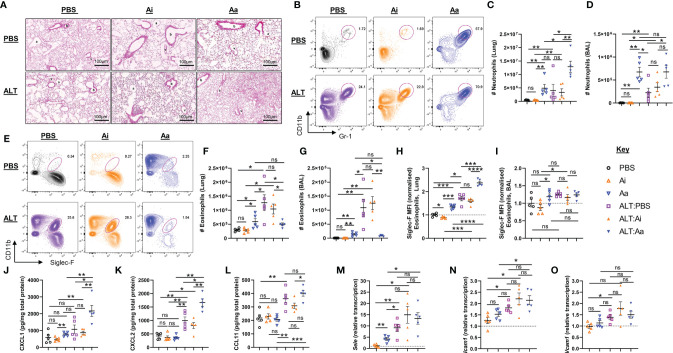
Granulocyte responses in the lung and airways following acute fungal allergen challenge in the context of enzymatic depletion of airway acetylcholine. Mice were dosed with Alternaria extract (ALT) or vehicle (PBS) and/or inactive AChE (Ai), active AChE (Aa) or vehicle (PBS) in the combinations indicated. For dosing regimen, see related **Supplemental Figure 4A**. **(A)** Representative H&E-stained lung sections. b, bronchiole; v, blood vessel; a, alveolus. **(B)** Representative flow cytometry plots for airway neutrophils from the indicated mouse strains and treatment groups. **(C)** Number of neutrophils in the lung. **(D)** Number of neutrophils in the airways as retrieved by bronchoalveolar lavage (BAL). **(E)** Representative flow cytometry plots for airway eosinophils from the indicated mouse strains and treatment groups. **(F)** Number of eosinophils in the lung. **(G)** Number of eosinophils in the airways, retrieved by BAL. Normalised mean fluorescence intensity (MFI) of **(H)** Siglec-F staining for lung eosinophils and **(I)** Siglec-F staining for airway eosinophils. Quantification of protein expression from total lung protein lysate for **(J)** CXCL1. **(K)** CXCL2. **(L)** eotaxin-1(CCL11). qRT-PCR analysis of **(M)** Sele (E-selectin), **(N)** Icam1 (intercellular cell adhesion molecule 1), **(O)** Vcam1 (Vascular cell adhesion protein 1) from total lung tissue. Transcript levels were normalized to reference genes peptidylprolyl isomerase A (Ppia) and eukaryotic translation elongation factor 2 (Eef2) and calculated as ratios of pooled PBS control treated group samples (relative transcript level = 1). MFI data are normalised to the mean of PBS control values. Numbers on the cytometry plots indicate proportion of the parent Live/CD45^+^ gate. Data points represent individual animals. Data are representative of 2 independent experiments with n = 5 mice per group. *p < 0.05, **p < 0.01, ***p < 0.001, ****p < 0.0001, ns, non-significant difference (p >0.05).

Intranasal administration of *Alternaria* to mice resulted in a moderate influx of neutrophils (**Figures 3A–D**) and pronounced eosinophilia in the lungs and airways after 48 hours, as anticipated (**Figures 3E–G**) (15, 16). When *Alternaria* was co-administered with enzymatically active AChE, elevated numbers of neutrophils were observed in the lungs (**Figures 3A–D**), but eosinophilia was strikingly reduced in both sites (**Figures 3E–G**). Inactive AChE had no effect on eosinophil or neutrophil numbers, indicating that the effects of the active enzyme were due to hydrolysis of ACh (**Figures 3B–G**). Treatment with active AChE alone also resulted in significantly increased numbers of neutrophils (**Figures 3B–D**) and a small increase in eosinophils (**Figures 3E–G**) relative to PBS and inactive AChE baseline controls. Of note, eosinophil numbers in active AChE-treated mice at baseline were lower than those in *Alternaria* + PBS/inactive AChE-exposed tissues.

Granulocytes in the lungs also showed some phenotypic alterations following the depletion of ACh (**Figures 3H, I** and **Supplemental Figures 4B–G**). Eosinophils in the lung tissue, but not the airways, of *Alternaria-*treated mice demonstrated enhanced expression of Siglec-F, typical of recruited inflammatory cells (23), and this was further enhanced by co-administration of active but not inactive AChE (**Figures 3H, I**). Exposure to active AChE alone also slightly enhanced eosinophil Siglec-F expression, but to a lesser extent than following *Alternaria* exposure (**Figure 3H**). Depletion of ACh resulted in reduced CD11b (Integrin alpha M) expression on the surface of eosinophils and neutrophils (**Supplemental Figures 4B–E**), wheareas expression of Gr-1 (Ly6C/Ly6G antigen) on airway neutrophils was enhanced by either *Alternaria* or depletion of ACh (**Supplemental Figures 4F**).

Given the striking alterations to inflammatory cell influx caused by the depletion of ACh, we investigated whether this was accompanied by an altered chemokine environment. The level of CXCL1 in lung extracts was slightly increased by exposure to active AChE alone but was greatly enhanced by *Alternaria* + active AChE (**Figure 3J**). CXCL2 was not affected by active AChE alone but was considerably enhanced by co-administration of *Alternaria* and active AChE (**Figure 3K**). In contrast, although eotaxin-1 was only elevated following exposure to *Alternaria*, its maximal level was unaffected by ACh depletion (**Figure 3L**). In all cases, co-administration of an inactive enzyme with *Alternaria* had no significant effect on chemokine levels in the pulmonary tissues.

As previous studies have indicated that endothelial cell adhesion molecules could be downregulated in response to cholinergic agonists during inflammation (24, 25), we also assessed whether hydrolysis of airway ACh affected their expression. Administration of *Alternaria* induced pronounced upregulation of E-Selectin (*Sele*) (**Figure 3M**) and modest increases in Intercellular adhesion molecule 1 (*Icam1*) (**Figure 3N**) and Vascular cell adhesion molecule 1 (*Vcam1*) (**Figure 3O**) transcripts in lung tissue, but these were not further affected by co-administration of either active or inactive AChE. A small increase in *Sele* was also observed when active AChE was administered alone, in the absence of *Alternaria* (**Figure 3M**).

### ILC2 Activation and Type 2 Cytokine Production in Response to *Alternaria* Allergen Extract is Profoundly Inhibited Following Enzymatic Depletion of Airway Acetylcholine

**Figure 4:**
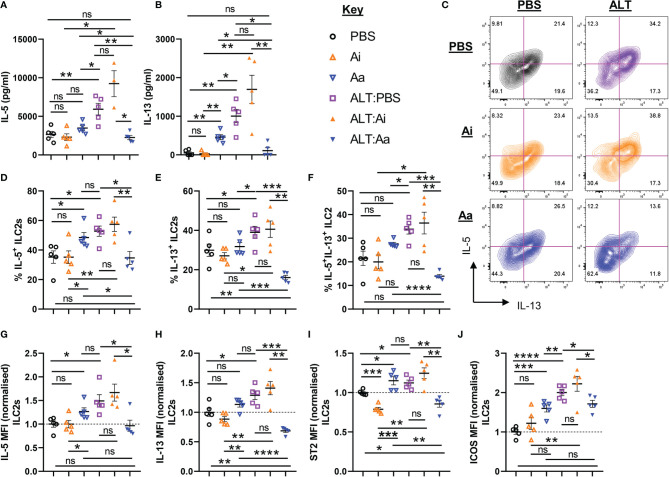
ILC2 responses following acute fungal allergen challenge in the context of enzymatic depletion of airway acetylcholine. Mice were dosed with Alternaria extract (ALT) or vehicle (PBS) and/or inactive AChE (Ai), active AChE (Aa) or vehicle (PBS) in the combinations indicated. For dosing regimen, see related **Supplemental Figure 4A**. **(A)** IL-5 and **(B)** IL-13 expression in the supernatant of total lung cells cultured with PMA/Ionomycin, as determined by ELISA. **(C)** Representative flow cytometry plots for IL-5 and IL-13 expression by pulmonary ILC2s from the indicated mouse strains and treatment groups. Numbers on the plots indicate proportion of the parent ILC2 population for each gate. **(D)** Proportion of IL-5^+^ ILC2s. **(E)** Proportion of IL-13^+^ ILC2s**. (F)** Proportion of IL-5^+^IL-13^+^ ILC2s. **(G–J)** Mean fluorescence intensity (MFI) of **(G)** IL-5 staining for IL-5^+^ ILC2s, **(H)** IL-13 staining for IL-13^+^ ILC2s, **(I)** ST2 staining for ILC2s, **(J)** ICOS staining for ILC2s. MFI data **(G–J)** are normalized to the mean of PBS control values. Data points represent individual animals. Data are representative of 2 independent experiments with n = 5 mice per group. *p < 0.05, **p < 0.01, ***p < 0.001, ****p < 0.0001, ns, non-significant difference (p >0.05).

As anticipated, administration of *Alternaria* resulted in increased production of IL-5 and IL-13 by total unseparated cells from lung tissue, but this was significantly reduced following non-selective depletion of airway ACh (**Figures 4A, B**). Active AChE treatment alone did not alter total IL-5 production, although IL-13 was increased (**Figure 4B**). Although ILC2-derived ACh did not appear to impact on these changes (**Figure 2**), we reasoned that ILC2 activity may still be dependent directly or indirectly on cholinergic signaling and therefore investigated this in more detail. Expression of IL-5 and IL-13 was increased by pulmonary ILC2s in response to *Alternaria*, with a significantly higher proportion of ILC2s expressing the cytokines and higher per-cell expression levels (**Figures 4C–H**). This was strongly suppressed by active but not inactive AChE (**Figures 4C–H**). Conversely, treatment with active AChE at baseline had the opposite effect, increasing IL-5 and IL-13 production by lung ILC2s relative to PBS and inactive AChE-treated controls (**Figure 4C–H**). Cytokine production by ILC2s reflected the activation status of the cells, as measured by ST2 and ICOS expression, which were enhanced in *Alternaria-*treated mice alone or with inactive AChE, and in active AChE only treated mice, but restricted following depletion of ACh with active enzyme in the context of *Alternaria* exposure (**Figures 4I, J** **)**.

### Alternative Activation of Pulmonary Macrophages is Inhibited by Depletion of Acetylcholine

Both non-selective depletion of ACh and loss of ILC2-specific capacity to synthesize ACh enhanced neutrophil chemoattractants and cellular influx into the lungs following exposure to *Alternaria*, indicating that neutrophil trafficking is sensitive to suppression by cholinergic signaling. We therefore examined the M1/M2 phenotypes of pulmonary macrophages following enzymatic depletion of ACh. We analyzed three macrophage populations: monocyte-derived airway macrophages (Mo-AMΦ), tissue resident alveolar macrophages (TR-AMΦ), and interstitial macrophages (IMΦ) in lung tissue (**Supplemental Figures 5A–E**).

**Figure 5:**
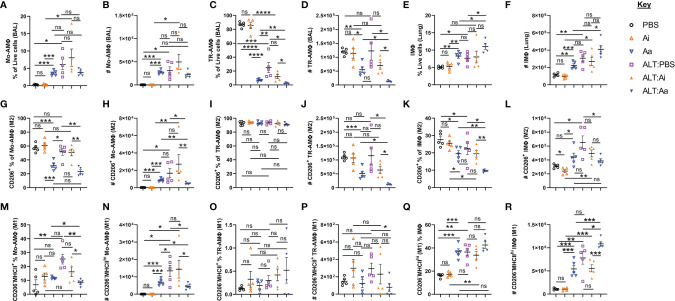
Alveolar and interstitial macrophage responses and alternative activation following acute fungal allergen challenge in the context of enzymatic depletion of airway acetylcholine. Mice were dosed with Alternaria extract (ALT) or vehicle (PBS) and/or inactive AChE (Ai), active AChE (Aa) or vehicle (PBS) in the combinations indicated. For dosing regimen, see related **Supplemental Figure 4A**. Monocyte-derived alveolar macrophages (Mo-AMΦ), tissue-resident alveolar macrophages (TR-AMΦ) and lung interstitial macrophages (IMΦ) were analysed for M1 (CD206^-^MHCII^hi^) and M2 (CD206^+^) phenotypes. **(A)** Mo-AMΦ as the proportion of the total live cell population in the airways. **(B)** Number of Mo-AMΦ in the airways. **(C)** TR-AMΦ as the proportion of the total live cell population in the airways. **(D)** Number of TR-AMΦ in the airways **(E)** IMΦ as the proportion of the total live CD45^+^ cell population in the lung. **(F)** Number of IMΦ in the lung. **(G)** M2 Mo-AMΦ as the proportion of total Mo-AMΦ in the airways. **(H)** Number of M2 Mo-AMΦ in the airways. **(I)** M2 TR-AMΦ as the proportion of total TR-AMΦ in the airways. **(J)** Number of M2 TR-AMΦ in the airways. **(K)** M2 IMΦ as the proportion of total IMΦ in the lung. **(L)** Number of M2 IMΦ in the lung. **(M)** M1 Mo-AMΦ as the proportion of total Mo-AMΦ in the airways. **(N)** Number of M1 Mo-AMΦ in the airways. **(O)** M1 TR-AMΦ as the proportion of total TR-AMΦ in the airways. **(P)** Number of M1 TR-AMΦ in the airways. **(Q)** M1 IMΦ as the proportion of total IMΦ in the lung. **(R)** Number of M1 IMΦ in the lung. CD206 and MHCII gating was carried out based on absence of expression in fluorescence minus one (FMO) controls. Data points represent individual animals. Data are representative of 2 independent experiments with n = 5 mice per group. *p < 0.05, **p < 0.01, ***p < 0.001, ****p< 0.0001, ns, non-significant difference (p >0.05).

Mice exposed to *Alternaria* alone or *Alternaria* + inactive AChE showed enhanced frequencies and numbers of Mo-AMΦ (**Figures 5A–B**), no alterations in overall numbers of TR-AMΦ, but reduced frequencies of these cells due to greatly enhanced neutrophil and eosinophil influx, (**Figures 5C, D**), and increased frequencies and numbers of total IMΦ in the lung (**Figures 5E, F**). *Alternaria* + active AChE treatment did not alter total Mo-AMΦ (**Figures 5A, B**) but reduced total TR-AMΦ (**Figures 5C, D**) and resulted in a non-significant trend toward increased lung IMΦ (**Figures 5E, F**). Treatment with inactive AChE alone did not alter macrophage populations relative to PBS only treated controls, however active AChE alone resulted in significantly more Mo-AMΦ (**Figure 5B**), less TR-AMΦ (**Figure 5D**) and more IMΦ (**Figure 5E**). Notably, *Alternaria* + active AChE treatment resulted in significantly less TR-AMΦ (**Figure 5D**) but more IMΦ (**Figure 5E**), compared to the active AChE alone.

A characterization of macrophage polarization phenotypes revealed that *Alternaria* exposure combined with ACh depletion reduced the numbers expressing mannose receptor c-type 1 (CD206) (**Figures 5G–L**), a specific indicator of the M2 phenotype (26). Conversely, active AChE treatment alone showed a tendency towards increased numbers of M2 Mo-AMΦ (**Figure 5H**) and IMΦ (**Figure 5L**) relative to PBS and inactive AChE-treated controls. To investigate whether this was correlated with enhancement of the M1 phenotype, we gated on CD206^-^MHCII^hi^ macrophages (**Supplemental Figure 5E**). Active AChE alone, and *Alternaria* treatment + PBS or inactive AChE increased numbers of M1 Mo-AMΦ, while active AChE treatment and *Alternaria* co-exposure decreased M1 Mo-AM and TR-AMΦ relative to *Alternaria* exposed controls (**Figures 5M–P**). However, M1-like IMΦ were increased significantly in *Alternaria* + active AChE treated mice, relative to increases observed in *Alternaria* + PBS and inactive AChE controls as well as active AChE only treated animals (**Figures 5Q, R**).

Cumulatively, these data indicate that cholinergic signaling is an important and complex regulator of immune responses, both at baseline during homeostasis and during fungal allergen-induced, allergic inflammation in the murine lung.

## Discussion

As we recently demonstrated that ILC2-derived ACh is required for optimal type 2 responses to parasitic infection, we sought to determine whether this also plays a role in allergic inflammation. *Rora* ^Cre+^ *Chat* ^LoxP^ mice showed enhanced airway neutrophilia following fungal allergen exposure, indicating that neutrophil influx is regulated in part by ILC2-derived ACh. This was further exacerbated by non-selective depletion of ACh, suggesting that there are multiple cellular sources of ACh which contribute to the suppression of neutrophil influx into the airways. We have not determined the precise sources of these signals, which could be neuronal, epithelial (9), or lymphoid (4).

A recent study showed that blockade of ACh synthesis by inhibition of choline uptake in the airways promoted neutrophilic inflammation and delayed the recovery of mice infected with influenza A (8), consistent with our current data. B cells, CD4^+^, and CD8^+^ T cells were identified as potential sources of ACh *via* the use of transgenic choline acetyltransferase (ChAT) reporter mice (8), and we have also identified ChAT expression in several lymphocyte lineages in the lungs (4). B cell-derived ACh has previously been demonstrated to inhibit neutrophil recruitment to the peritoneum in an LPS-induced model of sterile sepsis, and it was concluded that this resulted from suppression of expression of intercellular adhesion molecule 1 (ICAM-1) and vascular cell adhesion molecule 1 (VCAM-1) on vascular endothelial cells, signaling through mAChRs (24). This built upon other studies which indicated that ACh reduced expression of adhesion molecules and suppressed chemokine release from endothelial cells (25) and moreover suppressed expression of CD11b by neutrophils (27), in both cases signaling through the α7 nicotinic acetylcholine receptor (nAChR).

In our current study, no alteration in the expression of the adhesion molecules ICAM-1, VCAM-1, and E-selectin in lung tissue was observed after the depletion of ACh in the context of *Alternaria* induced airway inflammation, and CD11b expression on both neutrophils and eosinophils was suppressed rather than enhanced. However, depletion of ACh resulted in clearly enhanced expression of the neutrophil-attractant chemokines CXCL1 and CXCL2, and this was also evident in *Rora* ^Cre+^ *Chat* ^LoxP^ mice. We therefore conclude that ACh deficiency leads to alterations in chemokine expression, which are primarily responsible for the influx of neutrophils into the lungs in this model of allergic inflammation. However, depletion of ACh without exposure to *Alternaria* did enhance pulmonary E-Selectin (*Sele*) expression in addition to CXCL1, concomitant with enhanced neutrophilia, suggesting that ACh plays an important role in the restriction of neutrophil influx in the absence of an overt inflammatory stimulus. Previously, we have identified lung B cells as a significant source of non-neuronal ChAT and thus ACh synthesis in mice (4). Therefore, in line with previous studies (24, 28), it is possible that B cell production of ACh regulates neutrophil trafficking at homeostasis in the lung.

While neutrophilia was promoted, depletion of ACh also prevented the characteristic eosinophilia associated with *Alternaria* exposure, most likely due to restricted ILC2 activation and release of IL-13 and IL-5. These cytokines operate in concert with eotaxin to recruit eosinophils to the lungs (29, 30) and are also important in the maintenance of cellular viability (31, 32). Expression of Siglec-F is increased in eosinophils following lung inflammation (23) and has been proposed to regulate cell numbers *via* induction of apoptosis (33). In our current study, depletion of ACh resulted in enhanced expression of Siglec-F on lung eosinophils, and it is therefore possible that this contributes to impaired cell survival. Conversely, ACh depletion in the absence of *Alternaria* exposure slightly increased eosinophil numbers in the lung. This is most likely due to the activating effect that ACh depletion at baseline appeared to have on lung ILC2s.

Recent evidence demonstrated that synthesis of ACh was required for resolution of inflammation following respiratory viral infection, accompanied by increased numbers of ChAT^+^ lymphocytes in lung tissues (8). These lymphocytes were found in direct physical contact with pulmonary macrophages (8), suggesting that lymphocyte-derived ACh might play a role in driving macrophage polarization to an anti-inflammatory phenotype required for tissue repair (19, 34). Physical contact of cells is particularly relevant as the labile nature of ACh necessitates that intercellular signaling functions over relatively short distances. Tissue resident alveolar macrophages are the predominant leucocyte population present in the airways of healthy mice at homeostasis. Most of these cells are CD206^+^ and co-express additional markers of the M2 phenotype (26). In contrast, inflammation promotes the influx and development of monocyte-derived airway macrophages, characterized by expression of CD11b and Siglec-F (35–37). Depletion of ACh reduced the M2 macrophage pool by suppressing the proportion of monocyte-derived and interstitial macrophages that displayed an M2-like phenotype and decreasing the overall number of tissue-resident airway macrophages. This could result in part from the reduction of type-2 cytokines but also because ACh acts directly on macrophages to promote differentiation to the M2 state (38–40). Supportive of this, an increase in M1 macrophage marker expression was observed following both ILC2-specific ACh depletion and pan-ACh depletion. The enhanced expression of CXCL1 and CXCL2 following depletion of ACh in our model may therefore reflect a shift in macrophage phenotype from M2 to M1, although pericytes, endothelial, and mast cells are also important sources of neutrophil chemokines (18, 41, 42).

The role of specific muscarinic and nicotinic acetylcholine receptors in regulating pulmonary inflammation is complex. Muscarinic receptor subtype-deficient mice have been used to examine cigarette smoke-induced airway inflammation, with the conclusion that signaling through the M3 receptor was broadly pro-inflammatory, characterized by elevated neutrophils, macrophages, and lymphocytes in the airways and a corresponding increase in the expression of CXCL-1, CCL2, and IL-6, whereas signaling through the M1 and M2 mAChRs was generally anti-inflammatory, characterized by a reduction in cellular infiltration and chemokine levels (43). Tiotropium, a mAChR antagonist, which is functionally selective for the M3 subtype, is widely used to treat asthma and chronic obstructive pulmonary disease and to alleviate bronchoconstriction and mucus production, has been documented to reduce airway inflammation and remodeling (44, 45).

The effects of cholinergic signaling on ILC2s are similarly complex. Several reports indicate that α7 subtype-selective nAChR agonists reduce ILC2 effector function and airway hyperreactivity in an *Alternaria* allergic inflammation model (46, 47), whereas others indicate that broader, non-selective inhibition of nicotinic or muscarinic receptors can block activation (4, 5). A recent study demonstrated that inhibition of mAChR signaling with tiotropium attenuated ILC2 proliferation, type 2 cytokine production and eosinophilia in papain and IL-33-driven models of airway inflammation (48). Tiotropium did not inhibit cytokine production by isolated ILC2s *in vitro*, but did suppress IL-4 production by basophils, suggesting that ACh activates ILC2s indirectly *via* effects on basophils (48). Cholinergic signaling *via* mAChRs has also been demonstrated to stimulate ATP secretion in diverse tissues, suggesting another indirect route for activation of ILC2s by induction of IL-33 release (49, 50). Exemplifying the complexity of the effects of cholinergic signaling on ILC2s, enzymatic depletion of ACh had opposite effects on ILC2 activity in the lung dependent on exposure to *Alternaria*. Previously, we and others have shown that pulmonary ILC2s undergo significant alterations to ACh receptor expression following helminth and alarmin-induced activation, such as loss of the α7 inhibitory nAChR and upregulation of the excitatory M3 mAChR (4, 5). It is therefore possible that cholinergic signaling at homeostasis is required to restrict ILC2 activity, but supports effector responses following tissue-specific activation of the cells. Tissue signals, including IL-33, are known to regulate resident ILC2 at homeostasis and upon activation, increasing “basal activation” of the cells (51, 52). Therefore, inhibitory cholinergic signaling may serve an important role in the restriction of basal type 2 effector responses before the need for full activation of ILC2s in the context of exogenous challenge such as parasite infection and/or tissue damage.

Our data suggest that ACh has distinct effects associated with fungal allergen-induced pulmonary inflammation, on the one hand, promoting ILC2 cytokine production and eosinophilia, and on the other hand, inhibiting neutrophilia *via* suppression of CXCL1 and CXCL2 expression. ILC2-derived ACh plays a role in inhibition of neutrophil infiltration, potentially by directing macrophages towards an M2 phenotype, thus limiting expression of neutrophil chemoattractants.

## Materials and Methods

### Animals

This study was approved by the Animal Welfare Ethical Review Board at Imperial College London and was licensed by and performed under the UK Home Office Animals (Scientific Procedures) Act Personal Project Licence number 70/8193: ‘Immunomodulation by helminth parasites’. C57BL/6J and BALB/c mice, aged 6–8 weeks old, were purchased from Charles River. The *Rora* ^Cre+^ *Chat* ^Loxp^ mice used in this study were generated as previously described (4).

### Murine Model of Acute Fungal Allergen Exposure

Extracts of *A. alternata* were purchased as lyophilized protein extracts from Greer Laboratories (USA). Mice were anesthetized with aerosolized isofluorane before intranasal administration with 50 μg of *Alternaria* in a final volume of 50 µl of phosphate buffered saline without Ca^2+^ or Mg^2+^ (PBS, Sigma). Mice were exposed to a single dose of *Alternaria* for 24 or 48 h as indicated. Control animals were dosed with 50 μl of PBS on the same schedule. For co-administration of active or inactive AChE, 20 μg of either enzyme was mixed with 50 μg of *Alternaria* in PBS and the volume adjusted to 50 µl for the first dose, and 20 μg of the enzyme alone was administered in 50 μl of PBS for the second dose.

### Expression of Active and Inactive AChE From *N. brasiliensis*

*N. brasiliensis* AChE B was expressed in *Pichia pastoris* as a secreted protein and purified from culture supernatants as previously described (21). An enzymatically inactive form of the enzyme was generated *via* site-directed mutagenesis, changing the active site serine residue Ser-193 (Ser-200 in Torpedo AChE) to alanine (S193A), using the Quickchange site-directed mutagenesis kit (Stratagene) as previously described (22). The mutation was confirmed by sequencing before expression in *P. pastoris*, and purification confirmed that the enzyme was catalytically inactive. Proteins were passed through endotoxin removal columns (Pierce) and endotoxin removal was confirmed using a LAL Chromogenic Endotoxin Quantitation Kit (Pierce). Protein concentrations were determined using the Pierce Coomassie Plus (Bradford) Assay Kit (Thermo Scientific). AChE activity was determined by the method of Ellman with 1 mM acetylthiocholine iodide as the substrate in the presence of 1 mM 5,5’-dithiobis(2-nitrobenzoic acid) (DTNB) in 100 mM sodium phosphate pH 7.0 at 20°C. The reaction was monitored by measuring the absorbance at 412 nm, and the hydrolysis of acetylthiocholine iodide was calculated from the extinction coefficient of DTNB (53). One unit of AChE was defined as 1 μmol of substrate hydrolyzed per min at 20°C.

### Denaturing and Non-Denaturing Electrophoresis

Purified recombinant active and inactive enzymes were resolved by SDS-PAGE on 10% polyacrylamide gels followed by staining with Coomassie brilliant blue. The same preparations were resolved under non-denaturing conditions by electrophoresis in 7.5% polyacrylamide gels in Tris-Borate-EDTA buffer pH 8.0, and enzyme activity was assayed using the method of Karnovsky and Roots (54).

### Tissue Harvest and Preparation

For isolation of bronchoalveolar cells, the lungs were lavaged twice *via* the trachea in 2 ml of PBS with 0.2% BSA and 2 mM EDTA. Erythrocytes were lysed, leukocytes resuspended and counted. For lung single cell suspensions, lungs were perfused with PBS *via* injection into the heart before harvest, and lung leukocytes were isolated by dicing lung tissue and digesting for 1 h at 37°C with 300 U ml^−1^ of collagenase-II (Gibco) + 150 mg ml^−1^ of DNAse I (Sigma) in PBS without Mg^2+^ or Ca^2+^, followed by sample mashing through a 70 µm cell strainer into single-cell suspension, followed by red blood cell lysis.

### Flow Cytometry

Single cell suspensions were stained with fixable viability dyes (Invitrogen), then treated with rat anti-mouse CD32/CD16 (FcBlock, BD Biosciences), washed, and stained for extracellular markers using fluorophore conjugated monoclonal antibodies (eBioscience, Miltenyi Biotec, or Biolegend). For intracellular staining, cells were fixed for 30 min at room temperature, then permeabilized using the FoxP3/transcription factor staining buffer kit (eBioscience) and stained with fluorochrome-conjugated antibodies. Unstained samples and fluorescence minus one control were used as appropriate. Samples were analysed on a BD LSR Fortessa™ analyzer.

### Immunophenotyping of Leucocyte Populations

Unless otherwise stated, leucocyte populations were identified by flow cytometry by gating live cells, followed by single cell and CD45^+^ gating, and then using the following markers: ILC2: CD45^+^Lineage^-^CD127^+^GATA3^hi^ICOS^+^ST2^+^; CD4^+^ T cells: CD3^+^CD4^+^; neutrophils: CD11b^+^Siglec-F^-^GR-1^hi^CD11c^−/lo^; eosinophils: CD11b^+^Siglec-F^+^Gr-1^−/lo^CD11c^−^; tissue resident alveolar macrophages (TR-AMΦ): CD11b^−^F4/80^+^Siglec-F^+^CD11c^+^Gr-1^−^; monocyte-derived alveolar macrophages (MO-AMΦ): CD11b^+^F4/80^+^Siglec-F^+^CD11c^+^Gr-1^−^; interstitial lung macrophages (IMΦ): CD11b^+^F4/80^+^Siglec-F^-^CD11c^−/+^Gr-1^−^. CD206 was used as a marker of alternatively activated M2 macrophage subsets. Absence of CD206 expression (CD206^−^) and positive expression of MHCII (MHCII^hi^) were used to identify M1 macrophage subsets. The lineage panel consisted of antibodies against CD3, CD4, CD8, CD5, B220, CD19, TER119, CD49b, FcϵRI, CD11c, and Gr-1 (Ly6C/Ly6G). Scatter profiling of gated myeloid populations was also used to validate identity, as shown in **Supplemental Figure 1**.

### Intracellular Cytokine Capture

Single cell suspensions were diluted to 5× 10^6^ cells ml^−1^ in cDMEM (DMEM + 10% FCS, + 2 mM L-glutamine + 100 U ml^−1^ penicillin + 100 ug ml^−1^ streptomycin) and either stimulated for 4 h at 37°C/5% CO2 with 1 ug ml^−1^ PMA/100 ng ml^−1^ ionomycin with 1× brefeldin-A (GolgiPlug, BD Biosciences) + 1 μM monensin ((Sigma) or left unstimulated (golgi inhibitors alone). Samples were stained, fixed, and permeabilized as described, and intracellular staining for Fc receptor blocking followed by fluorophore conjugated mAbs against IL-5 and IL-13 was performed in permeabilization buffer.

### RT-qPCR

Total lung tissue was homogenized using a Tissuelyser II (Qiagen). Total RNA was extracted by TRIzol/chloroform phase-separation, DNAse-1 treated, then 1 μg of RNA was reverse transcribed using the iScript cDNA synthesis kit (Biorad). RT-qPCR reactions were carried out using the PowerUp SYBR Green Mix (ThermoFisher) in an ABI 7500 Fast Real-time PCR thermocycler (Applied Biosystems). RT-qPCR reactions were run in triplicate, with no template and no RT controls. Relative expression of *Ccl11, Cxcl1*, and *Cxcl2* was calculated by the comparative cycle threshold (Ct) method (2^−ΔΔCT^) using *Actb*, *Hprt*, and *Gapdh* as reference genes. *Eef2* and *Ppia* were used as the reference genes for calculating *Icam1, Vcam1*, and *Sele* expression. Primer sequences used for RT-qPCR were as follows:

***Nos2*: F:** 5’- CCGGCAAACCCAAGGTCTAC-3’, **R:** 5’- CTGCTCCTCGCTCAAGTTCA-3’.

***Arg1*: F:** 5’-AAAGGCCGATTCACCTGAGC -3’, **R:** 5’- CTGAAAGGAGCCCTGTCTTGTA-3’.

***Mrc1*: F:** 5’-GGAGGGTGCGGTACACTAAC-3’, **R:** 5’-TCAGTAGCAGGGATTTCGTCTG-3’.

***Ccl11*: >F:** 5’-TGGCTCACCCAGGCTCCATC-3’, **R:** 5’-TCTCTTTGCCCAACCTGGTCTT-3’.

***Cxcl1*: >F:** 5’-ACCCAAACCGAAGTCATAGCCA -3’, **R:** 5’-TCAGAAGCCAGCGTTCACCA-3’.

***Cxcl2*: >F:** 5’-TCCAAAAGATACTGAACAAAGGCAA-3’, **R:** 5’-ATCAGGTACGATCCAGGCTTCC-3’.

***Icam1*: F:** 5’-AGCTCGGAGGATCACAAACG-3’, **R:** 5’- TCCAGCCGAGGACCATACAG-3’.

***Sele*: F:** 5’-CCCAGTGCTTCTGGACCTTT-3’, **R:** 5’- TTCACAGCTGAACACGTGGG-3’.

***Vcam1*: F:** 5’-CGACCTTCATCCCCACCATT-3’, **R:** 5’-GGGGGCAACGTTGACATAAAG-3’.

***Eef2*: F:** 5’-CCCCAACATTCTCACCGACA-3’, **R:** 5’-AGAGAGCGCCCTCCTTAGTA-3’

***Ppia*: F:** 5’- GCATACAGGTCCTGGCATCT-3’, **R:** 5’- ATGCTTGCCATCCAGCCATT-3’

***Gapdh*: >F:** 5’-GTCATCCCAGAGCTGAACGG-3’, **R:** 5’-TACTTGGCAGGTTTCTCCAGG-3’.

***Actb*: >F:** 5’-TTCCTTCTTGGGTATGGAATCCT-3’, **R:** 5’-TTTACGGATGTCAACGTCACAC-3’.

***Hprt*: F:** 5’-ACAGGCCAGACTTTGTTGGA-3’, **R:** 5’-ACTTGCGCTCATCTTAGGCT-3’.

### Enzyme-Linked Immunosorbent Assay

Protein lysates were made by snap freezing the small right lung lobe directly after tissue harvest. Harvested lung samples were stored at −80°C and homogenised in PBS with an electric homogeniser. Samples were centrifuged and the supernatant collected. Samples were diluted to a final concentration of 50 mg of lung tissue ml^−1^ for use. Total lung cells were cultured at a density of 5 × 10^6/^ml for 12 h with 12.5 ng/ml PMA and 125 ng/ml Ionomycin, before collecting culture supernatants and centrifuging to clear residual cells, followed by freezing at −20°C until analysis. Proteins were quantified using commercial ELISA kits according to the instructions of the manufacturer (Duoset, R&D). For CXCL1 and CXCL2 detection, results were obtained on a Tecan Sunrise 96-well Microplate reader F039300 with Tecan Magellan Anlaysis Software V7.2 software. For Eotaxin-1 (Ccl11), IL-5 and IL-13, results were obtained on a FLUOStar Omega microplate reader.

### Histology

Lung tissue was harvested and fixed in 4% paraformaldehyde overnight at 4°C. Tissue was paraffin embedded, sectioned and stained with hematoxylin and eosin (H&E) according to standard techniques.

### Statistical Analysis

Flow cytometry data was analyzed using FlowJo software (Treestar). Graphs and statistical tests were carried out using Graphpad prism software (Graphpad). The normality of data distribution was analyzed by the Shapiro–Wilk test. Parametric data were analyzed by Welch’s t-test, non-parametric data were analyzed by Mann–Whitney-U test. Data represent mean ± SEM unless otherwise stated. Statistical significance between groups is indicated as *p <0.05, **p <0.01, ***p <0.001, ****p <0.0001, n.s = non-significant difference (*p >*0.05).

## Data Availability Statement

The raw data supporting the conclusions of this article will be made available by the authors, without undue reservation.

## Ethics Statement

The animal study was reviewed and approved by the Animal Welfare Ethical Review Board at Imperial College London and was licensed by and performed under the UK Home Office Animals (Scientific Procedures) Act Personal Project Licence number 70/8193: ‘Immunomodulation by helminth parasites’.

## Author Contributions

Conception and design: LR, RB, CS, and MS. Experimental work: LR, RB, MW, CS, DP, MD, and MS. Analysis and interpretation: LR, RB, MW, CS, and MS. Provision of Resources: LR, GL, KG, SB, BR, VQ, WH, and MS. Drafting the initial manuscript: LR and MS. Drafting the revised manuscript: LR and MS. All authors listed have made a substantial, direct, and intellectual contribution to the work and approved it for publication.

## Funding

This work was funded by a project grant to MS from the BBSRC (BB/R015856/1) and a PhD studentship to LR from the Wellcome Trust (097011).

## Conflict of Interest

The authors declare that the research was conducted in the absence of any commercial or financial relationships that could be construed as a potential conflict of interest.

## Publisher’s Note

All claims expressed in this article are solely those of the authors and do not necessarily represent those of their affiliated organizations, or those of the publisher, the editors and the reviewers. Any product that may be evaluated in this article, or claim that may be made by its manufacturer, is not guaranteed or endorsed by the publisher.
