# Supplementary material for: Differential Regulation of Allergic Airway Inflammation by Acetylcholine
Source: Front Immunol. 2022 May 27;13:893844. doi: 10.3389/fimmu.2022.893844 (PMC9196131; doi:10.3389/fimmu.2022.893844)
Supplement: Supplementary file 1 [file DataSheet_1.docx]

**Supplemental Material**


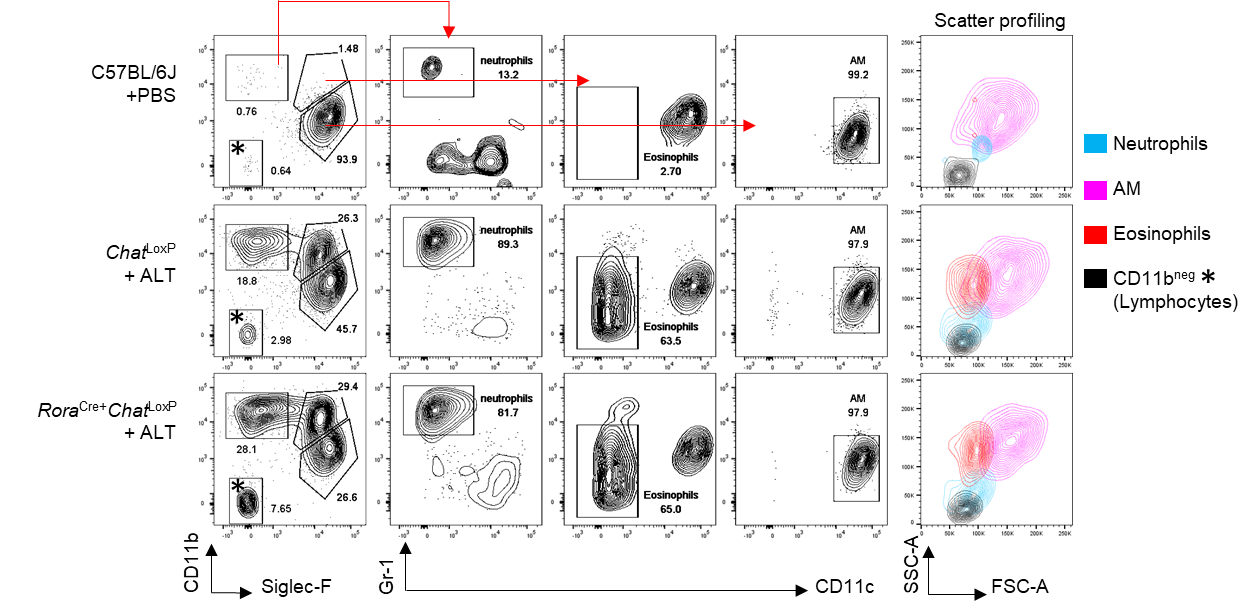


**Supplemental Figure 1. Representative airway leucocyte gating strategy for PBS and *Alternaria*-exposed C57BL/6J, *Chat*^LoxP^ and *Rora*^Cre+^*Chat*^LoxP^ mice (related to Figure 1).** Populations are pre-gated as live single cells, CD45^+^. Cell scatter profiling/back-gating of indicated populations was carried out to validate population identity based on expected forward (FSC) and Side (SSC) scatter parameters. Based on negative CD11b expression and small FSC/SSC profile, CD11b^-^ leucocytes (marked with *****) in the airways were defined as lymphocytes. AM = alveolar macrophages.


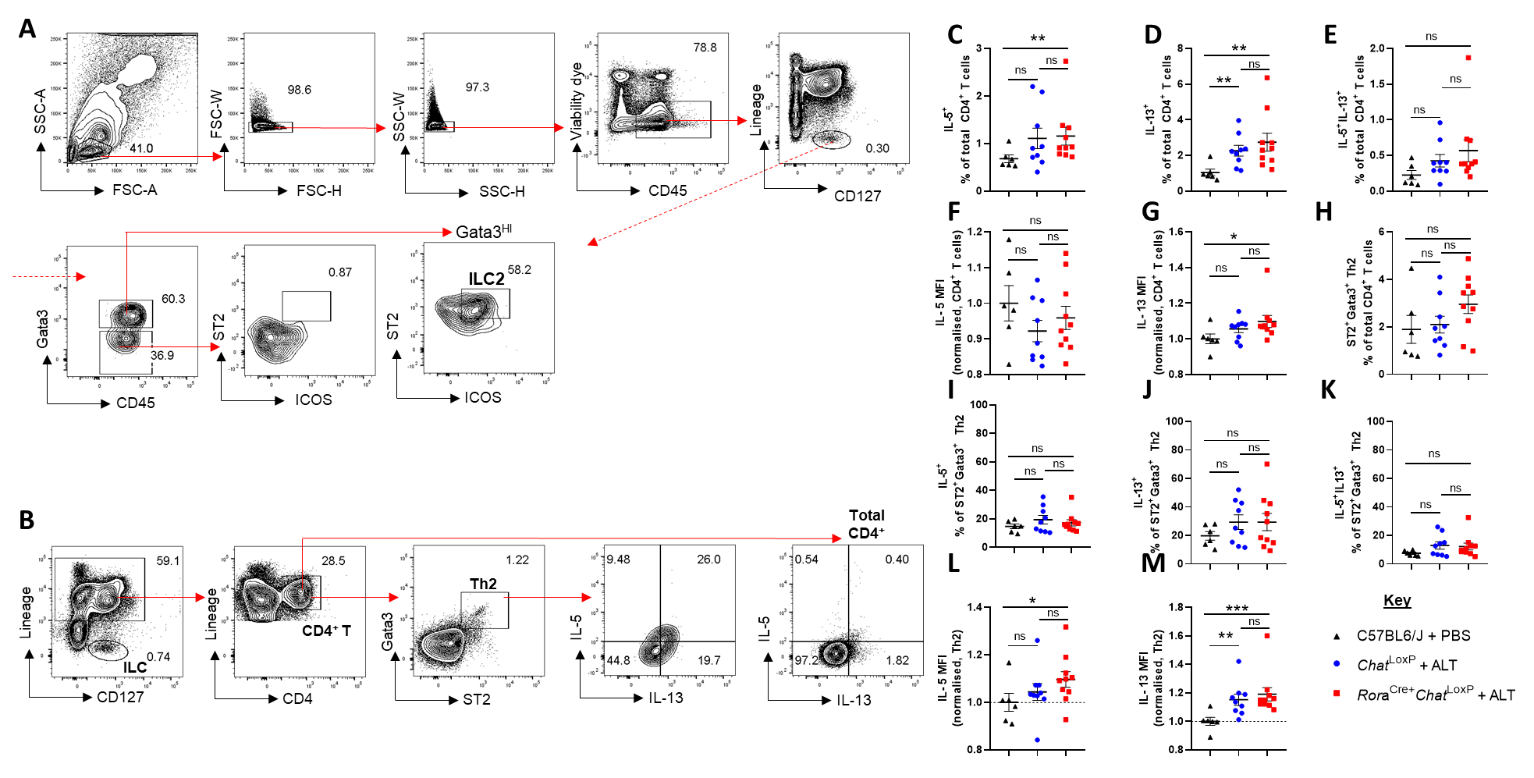


**Supplemental Figure 2. ILC2s gating strategy and pulmonary CD4^+^ T cell responses of *Chat*^LoxP^ and *Rora*^Cre+^*Chat*^LoxP^ mice following exposure to *Alternaria* (related to Figure 2). A.** Representative gating strategy for lung ILC2s. ST2^+^ICOS^+^ gate for Gata3^hi^ ILC2s was set based on negative expression in the Gata3^lo,^ non-ILC2s Lineage^-^CD127^+^ ILC population, as shown. **B.** Representative gating strategy for IL-5 and IL-13 expression by total CD4+ T cells and Gata3^+^ST2^+^ Th2 cells in the lung. Cells were pre-gated as live, CD45+ single cells with low forward and side scatter. **C**. Proportion of IL-5^+^ total CD4^+^ T cells. **D.** Proportion of IL-13^+^ total CD4+ T cells. **E.** Proportion of IL-5^+^IL-13^+^ total CD4^+^ T cells**. F.** Mean fluorescence intensity (MFI) of IL-5 staining for IL-5^+^ total CD4^+^ T cells. **G.** MFI of IL-13 staining for IL-13^+^ total CD4^+^ T cells. **H. I.** Proportion of IL-5^+^ Th2 cells. **J.** Proportion of IL-13^+^ Th2 cells. **K.** Proportion of IL-5^+^IL-13^+^ Th2 cells **L.** MFI of IL-5 staining for IL-5^+^ Th2 cells **M.** MFI of IL-13 staining for IL-13^+^ Th2 cells. MFI data **F-G** and **L-M** are normalised to the mean of PBS control values. Numbers on the flow cytometry plots indicate proportion of the indicated parent population. Data points represent individual animals. Data show pooled data points from 2 independent experiments with n=3 C57BL/6J mice per group and n=4-5 *Rora*^Cre+^*Chat*^LoxP^ and *Chat*^LoxP^ mice per experiment. *p<0.05, **p<0.01, ***p<0.001, ns = non-significant difference (*p*>0.05).


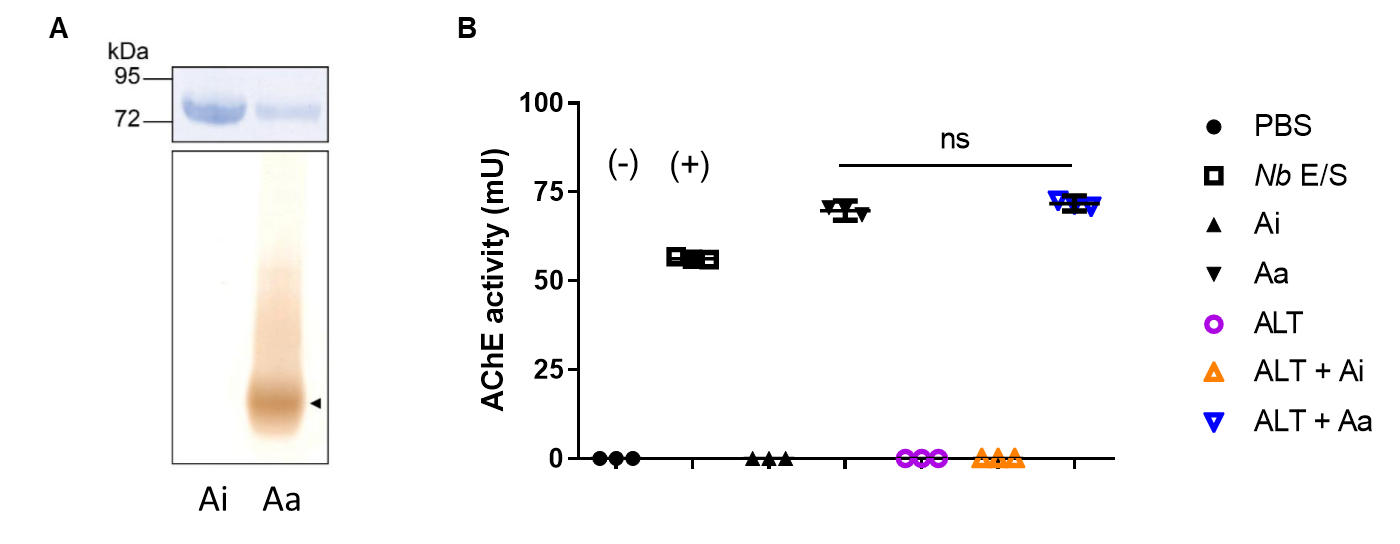


**Supplemental Figure 3. In-gel acetylcholinesterase activity and Ellman’s assay for active and inactive enzyme preparations (related to Figures 3, 4 & 5). A. Top:** Coomassie stained SDS-polyacrylamide gel of purified inactive (Ai) and active (Aa) AChE. Molecular mass standards are shown. **Bottom**: Staining of purified inactive and active preparations for AChE activity following resolution by non-denaturing PAGE. Brown staining indicates presence of AChE activity and black arrow denotes enzyme migration. **B.** Ellman’s assay for detection of AChE activity, shown in milliunits. Equimolar concentrations of active and inactive enzyme preparations were utilised alone or in combination with *Alternaria alternata* extract (ALT), mixed at the same concentrations as administered to mice*. Nippostrongylus brasiliensis* excretory/secretory products (*Nb* E/S, +) and PBS (-) were used as a positive and negative controls for AChE activity (+). ns = non-significant difference (*p*>0.05). Assay run with technical triplicates for each sample.

**
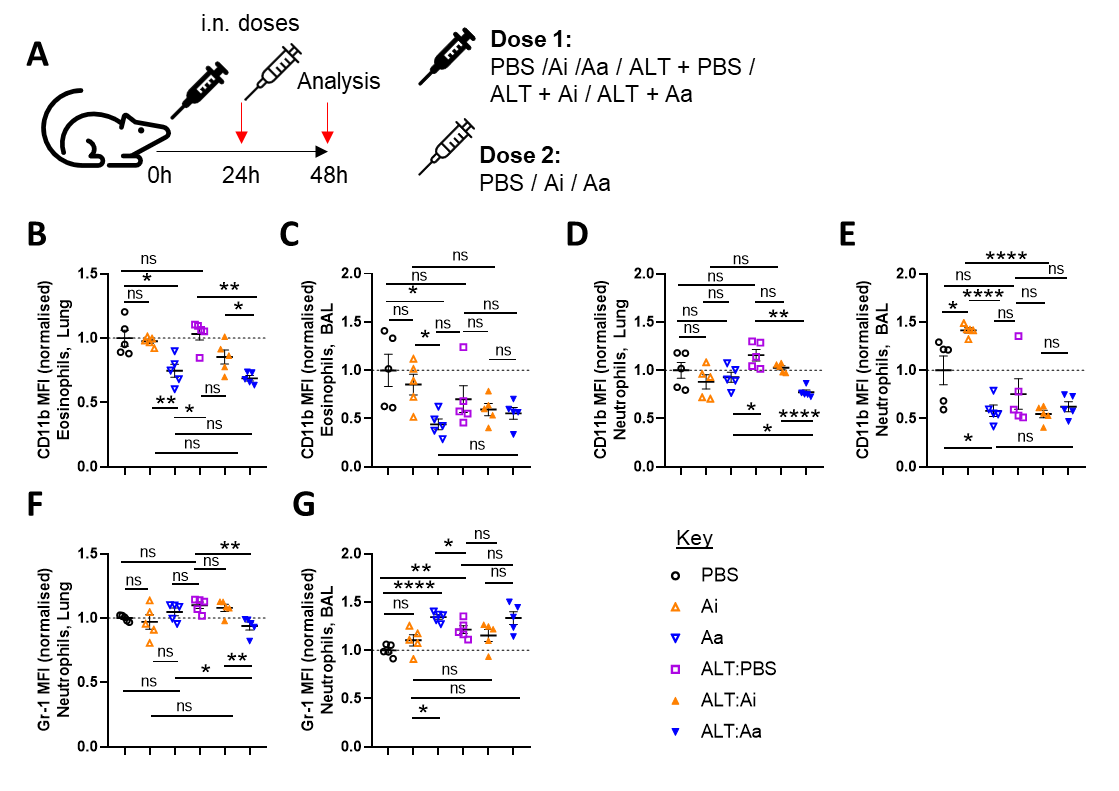
**

**Supplemental Figure 4. Intranasal dosing schematic and surface marker expression analysis of eosinophils and neutrophils following exposure to *Alternaria* and AChE (related to Figure 3). A.** BALB/c mice were intranasally dosed with PBS alone (PBS), *Alternaria alternata* allergen extract (ALT) in PBS alone (ALT:PBS), or co-dosed with ALT and inactive AChE (ALT:Ai) or ALT and active AChE (ALT:Aa). A second dose of PBS, Ai or Aa was given 24 hours after the initial dose to the relevant groups. Sample harvest and analysis was conducted 48 hours after the initial dose. Mean fluorescence intensity (MFI) of **B.** CD11b staining for lung eosinophils. **C.** CD11b staining for airway eosinophils. **D.** CD11b staining for lung neutrophils. **E.** CD11b staining for airway neutrophils. **F.** Gr-1 staining for lung neutrophils**. G.** Gr-1 staining for airway neutrophils**.** MFI data are normalised to the mean of PBS control values. Data points represent individual animals. Data are representative of 2 independent experiments with n=5 mice per group. *p<0.05, **p<0.01, ****p<0.0001, ns = non-significant difference (*p*>0.05).

**
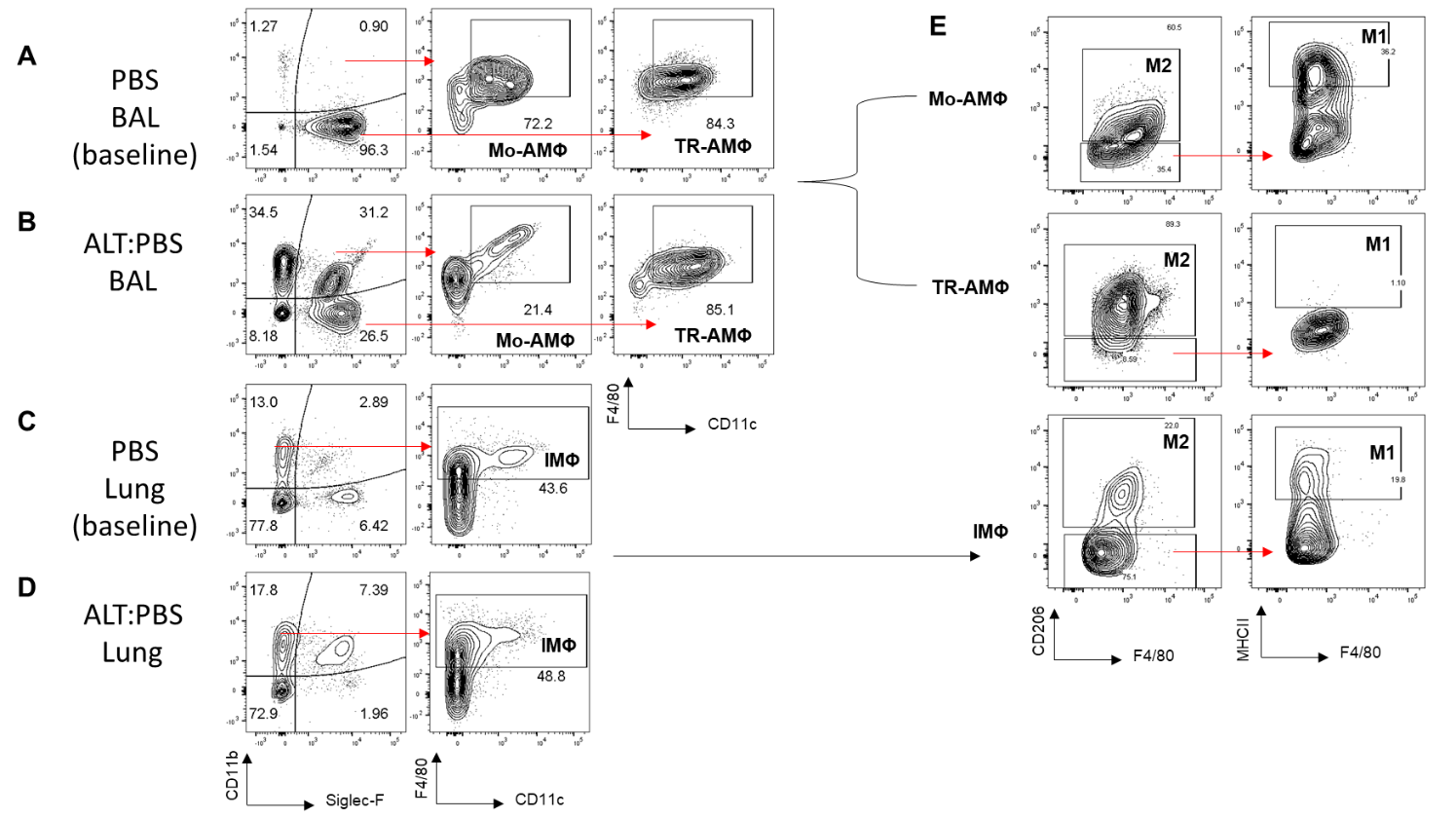
**

**Supplemental Figure 5. Representative gating strategies for alveolar and interstitial murine macrophage populations at baseline and following exposure to *Alternaria* (related to Figure 5).** Representative gating for monocyte-derived alveolar macrophages (Mo-AMΦ) and tissue-resident alveolar macrophages (TR-AMΦ) in the airways of **A.** PBS only treated control animals (baseline) and **B.** mice treated with *Alternaria alternata* allergen extract (ALT), and interstitial macrophages (IMΦ) in lung tissue of **C.** PBS only treated controls and **D.** ALT treated mice. **E**. M1 and M2 macrophages were identified based on CD206 and MHCII expression. Numbers on the flow cytometry plots indicate proportion of the indicated parent population. Populations are pre-gated as live single cells.
